# Functional Sites of Ribosome Modulation Factor (RMF) Involved in the Formation of 100S Ribosome

**DOI:** 10.3389/fmolb.2021.661691

**Published:** 2021-05-03

**Authors:** Hideji Yoshida, Hideki Nakayama, Yasushi Maki, Masami Ueta, Chieko Wada, Akira Wada

**Affiliations:** ^1^Department of Physics, Osaka Medical and Pharmaceutical University, Takatsuki, Japan; ^2^Bio Industry Business Department, Rapica Team, HORIBA Advanced Techno, Co., Ltd., Kyoto, Japan; ^3^Yoshida Biological Laboratory, Kyoto, Japan

**Keywords:** ribosome modulation factor, active sites, *Escherichia coli*, stress response, 100S ribosome

## Abstract

One of the important cellular events in all organisms is protein synthesis, which is catalyzed by ribosomes. The ribosomal activity is dependent on the environmental situation of the cell. Bacteria form 100S ribosomes, lacking translational activity, to survive under stress conditions such as nutrient starvation. The 100S ribosome is a dimer of two 70S ribosomes bridged through the 30S subunits. In some pathogens of gammaproteobacteria, such as *Escherichia coli*, *Yersinia pestis*, and *Vibrio cholerae*, the key factor for ribosomal dimerization is the small protein, ribosome modulation factor (RMF). When ribosomal dimerization by RMF is impaired, long-term bacterial survival is abolished. This shows that the interconversion system between active 70S ribosomes and inactive 100S ribosomes is an important survival strategy for bacteria. According to the results of several structural analyses, RMF does not directly connect two ribosomes, but binds to them and changes the conformation of their 30S subunits, thus promoting ribosomal dimerization. In this study, conserved RMF amino acids among 50 bacteria were selectively altered by mutagenesis to identify the residues involved in ribosome binding and dimerization. The activities of mutant RMF for ribosome binding and ribosome dimerization were measured using the sucrose density gradient centrifugation (SDGC) and western blotting methods. As a result, some essential amino acids of RMF for the ribosomal binding and dimerization were elucidated. Since the induction of RMF expression inhibits bacterial growth, the data on this protein could serve as information for the development of antibiotic or bacteriostatic agents.

## Introduction

In the exponential growth phase of bacteria, several proteins are synthesized by a massive amount of active ribosomes through four stages: initiation, elongation, termination, and recycling (Korostelev et al., 2008). However, under stress conditions such as nutrient starvation, ribosomal biosynthesis is repressed, and protein synthesis is suppressed by the formation of inactive 100S ribosomes (Wada et al., 1995; Yoshida and Wada, 2014). The 100S ribosome has no translational activity and is a dimer of two 70S ribosomes, which are bound via their 30S subunits (Kato et al., 2010; Ortiz et al., 2010; Polikanov et al., 2012). Ribosomal dimerization involves a small (Mr = 6,507) and basic (pI = 11.3) ribosome modulation factor (RMF). RMF has also been reported to be related to the resistance of the cell to heat stress (Niven, 2004), acid stress (El-Sharoud and Niven, 2005), and osmotic stress (Garay-Arroyo et al., 2000). In *Escherichia coli*, the expression of RMF is positively regulated by the stringent response alarmone (p)ppGpp (guanosine-3′,5′-bisdiphosphate or guanosine pentaphosphate) (Izutsu et al., 2001), the carbon source-sensing cAMP–cAMP receptor protein complex (cAMP-CRP) (Shimada et al., 2013), and the transcription factors stimulating biofilm formation such as McbR, RcdA, and SdiA (Yoshida et al., 2018). Another protein factor expressed during the stationary phase, hibernation promoting factor (HPF, also known as YhbH, Mr = 10,732), also binds to ribosomes and promotes 100S ribosome formation (Maki et al., 2000; Ueta et al., 2005, 2008). When the environmental conditions improve, RMF and HPF are immediately released from 100S ribosomes, which dissociate into active 70S ribosomes (Yamagishi et al., 1993; Aiso et al., 2005). Under stress conditions, ribosomes may also be inactivated by binding to the cold shock protein pY, also known as RaiA (ribosome-associated inhibitor A) or YfiA (Mr = 12,766) (Maki et al., 2000; Pietro et al., 2013). YfiA is an HPF paralog with 40% sequence homology and inhibits the formation of 100S ribosomes by blocking the binding of RMF and HPF to the active ribosome (Ueta et al., 2005). These systems of translational regulation are especially important for the survival of wild bacteria in harsh environments. We term the stage of inactivating ribosome “hibernation stage” in the ribosome cycle (Yoshida et al., 2002).

In some gammaproteobacteria, such as *E. coli*, RMF and HPF regulate 100S ribosome formation. Many other bacteria, such as *Thermus thermophilus*, do not express RMF and rely on the long HPF type (Mr = 21,550) for the 100S ribosome formation (Ueta et al., 2010, 2013; Tagami et al., 2012; Puri et al., 2014). The long HPF has a molecular weight approximately twice as high as that of the HPF expressed in gammaproteobacteria. In virtually all bacteria, except for gammaproteobacteria, 100S ribosomes are only formed by long HPF. Thus, bacteria have two distinct types of 100S ribosomes formed by RMF or long HPF. Structural studies based on cryo-electron microscopy (cryo-EM) showed that in the 100S ribosome of *Bacillus subtilis* and *Staphylococcus aureus*, the two 70S ribosomes are bridged by the C-terminus of long HPF (Beckert et al., 2017; Khusainov et al., 2017). On the other hand, RMF or HPF is not directly involved in the formation of the 100S ribosome in *E. coli* (Beckert et al., 2018). On the basis of these findings, it is thought that in *E. coli*, the formation of the 100S ribosome occurs via RMF-induced conformational changes in the 30S subunits, resulting in dimerization. Therefore, it is assumed that RMF contains functional sites contributing to ribosome binding and dimerization. The identification of these sites was the main purpose of the current study. Since many enteropathogenic bacteria, such as *Yersinia pestis* and *Vibrio cholerae*, express RMF for survival in harsh environments, these data on the functionally important sites of RMF could serve as information for the development of antibiotic or bacteriostatic agents.

## Materials and Methods

### Bacterial Strains and Growth

The bacterial strains and plasmids used in this work are listed in Table 1. First, a pRham plasmid carrying the intact *rmf* and *hpf* genes of *E. coli* (pRham-01) was constructed, which induced the expressions of RMF and HPF together by rhamnose. Next, the *rmf* gene in pRham-01 was replaced by gene versions carrying terminal deletions or point mutations (pRham-02∼28). The mutations of *rmf* gene are confirmed by the use of Applied Biosystems 3130 Genetic Analyzer. These multicopy plasmids were transformed into the *E. coli* strain, YB1005, obtained by deleting *rmf*, *hpf*, and *yfiA* genes from the wild-type strain (W3110). Overnight 1-mL precultures were obtained in medium E containing 2% polypeptone and were supplemented with 0.5% glucose at 37°C with shaking at 120 cycles per min (Vogel and Bonner, 1956). Medium E contains MgSO_4_, citric acid, K_2_HPO_4_, and NaNH_4_HPO_4_, in which *E. coli* can efficiently form 100S ribosomes under stress conditions. Mass cultures were performed in 200 mL of the same medium at 37°C with shaking at 120 cycles per min. To induce gene expression from plasmids, 0.8 mM rhamnose was preliminarily added to the medium of mass culture. The cells were harvested 2.5, 6, 9, and 24 h after the start of mass culture for subsequent analysis by sucrose density gradient centrifugation (SDGC) and western blotting. To confirm the expressions of mutant RMF from the plasmids, 0.8 mM rhamnose was added to the medium of mass culture at 4 h after inoculation, and cells were harvested 1 h later.

**TABLE 1:** *E. coli* strain and plasmids used in this work.

Strain or plasmid	Genotype	References
**Strain**		
YB1005	W3110 *Δrmf, Δhpf, ΔyfiAr::Km*	*Genes to Cells* (2013) 18, 554.
**Plasmids**		
pRham	Expression vector under control of rhaP_*BAD*_ promoter, Amp^*r*^	*J. Bacteriol.* (1998) 180, 1277.
pRham-01	pRham-based vector for expression of intact RMF and HPF	This study
pRham-02	pRham-01-derived vector for expression of RMF(ΔN5) and HPF	This study
pRham-03	pRham-01-derived vector for expression of RMF(ΔN10) and HPF	This study
pRham-04	pRham-01-derived vector for expression of RMF(ΔN15) and HPF	This study
pRham-05	pRham-01-derived vector for expression of RMF(ΔC5) and HPF	This study
pRham-06	pRham-01-derived vector for expression of RMF(ΔC10) and HPF	This study
pRham-07	pRham-01-derived vector for expression of RMF(ΔC15) and HPF	This study
pRham-08	pRham-01-derived vector for expression of RMF(R3A) and HPF	This study
pRham-09	pRham-01-derived vector for expression of RMF(K5A) and HPF	This study
pRham-10	pRham-01-derived vector for expression of RMF(R11A) and HPF	This study
pRham-11	pRham-01-derived vector for expression of RMF(G16A) and HPF	This study
pRham-12	pRham-01-derived vector for expression of RMF(G23A) and HPF	This study
pRham-13	pRham-01-derived vector for expression of RMF(C29A) and HPF	This study
pRham-14	pRham-01-derived vector for expression of RMF(P30A) and HPF	This study
pRham-15	pRham-01-derived vector for expression of RMF(C29A, P30A) and HPF	This study
pRham-16	pRham-01-derived vector for expression of RMF(W40A) and HPF	This study
pRham-17	pRham-01-derived vector for expression of RMF(G43A) and HPF	This study
pRham-18	pRham-01-derived vector for expression of RMF(W44A) and HPF	This study
pRham-19	pRham-01-derived vector for expression of RMF(R45A) and HPF	This study
pRham-20	pRham-01-derived vector for expression of RMF(G43A, W44A, R45A) and HPF	This study
pRham-21	pRham-01-derived vector for expression of RMF(E10A) and HPF	This study
pRham-22	pRham-01-derived vector for expression of RMF(I21A) and HPF	This study
pRham-23	pRham-01-derived vector for expression of RMF(T33A) and HPF	This study
pRham-24	pRham-01-derived vector for expression of RMF(M48A) and HPF	This study
pRham-25	pRham-01-derived vector for expression of RMF(ΔE10) and HPF	This study
pRham-26	pRham-01-derived vector for expression of RMF(ΔI21) and HPF	This study
pRham-27	pRham-01-derived vector for expression of RMF(ΔT33) and HPF	This study
pRham-28	pRham-01-derived vector for expression of RMF(ΔM48) and HPF	This study

*RMF (gene name: rmf): Key factor for ribosomal dimerization, HPF (gene name: hpf): Supportive factor for stabilization of ribosomal dimer, YfiA (gene name: yfiA): Inhibitive factor for ribosomal dimerization.*

### Measurement of Ribosome Profiles

The harvested *E. coli* cell pellets were suspended in an association buffer [100 mM CH_3_COONH_4_, 15 mM (CH_3_COO)_2_Mg.4H_2_O, 20 mM Tris–HCl at pH 7.6, and 6 mM 2-mercaptoethanol] and then vortexed with an approximately equal volume of glass beads (212–300 μm; Sigma-Aldrich Co., United States). The homogenate was centrifuged at 13,000 × *g* for 10 min at 4°C. The supernatant was layered on top of a 5–20% linear sucrose density gradient in association buffer and was centrifuged in an SW 41 Ti rotor (Beckman, United States) at 285,000 × *g* for 1.5 h at 4°C. After centrifugation, the absorbance of the sucrose gradient was measured at 260 nm using a UV-1800 spectrophotometer (Shimadzu, Japan).

### Western Blot Analysis

The 70S and 100S ribosomal fractions in the solution after SDGC were collected using a mechanical fraction collector (AC-5700P, ATTO Co., Ltd., Japan). Proteins in each fraction were precipitated with 10% trichloroacetic acid (TCA), separated by 16% tricine-sodium dodecyl sulfate-polyacrylamide gel electrophoresis (Tricine SDS-PAGE) (Schagger and von Jagow, 1987), and transferred to PVDF membranes (Immobilon-FL transfer membrane, Millipore, German). RMF and HPF were recognized by the corresponding rabbit antisera and detected with ECF substrate (GE Healthcare, United States) using a Typhoon FLA 9000 imager (GE Healthcare, United States).

## Results

### Conserved Amino Acids in RMF

**FIGURE 1:**
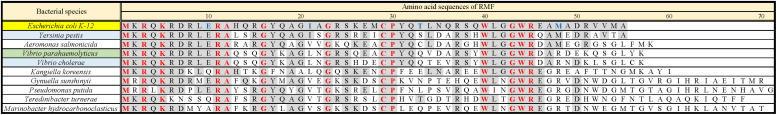
Alignment of RMF amino acid sequences between 10 species of gammaproteobacteria. Completely conserved amino acids are represented in red. RMF of the *E. coli* strain used in this work is shown at the top with a yellow background. The representative pathogens, *Yersinia pestis* and *Vibrio cholerae*, are indicated by blue background. The structure of RMF (PDB ID: 2JRM) in Figure 6 and Supplementary Figure 6 is that of *Vibrio parahaemolyticus*, which is shown with a green background. Incompletely conserved amino acids were used as references and are shown in blue characters in the *E. coli* sequence (top line). The alignment from 50 bacteria is shown in Supplementary Figure 1.

Ribosome modulation factor, a key factor for the formation of 100S ribosome, is highly conserved among gammaproteobacteria. Figure 1 and Supplementary Figure 1 show the alignments of amino acid sequences of RMF from 10 and 50 bacteria, respectively. In many pathogens, such as *Yersinia pestis* and *Vibrio cholerae* (shown by blue backgrounds), RMF ensures survival in harsh environments. The RMF of *E. coli* consists of 55 amino acids, as indicated by the yellow background in the top line. Thirteen amino acids (red characters) are completely conserved among these 50 bacteria and comprise basic amino acids such as arginine (R) and lysin (K), which are expected to interact with rRNA. The conserved amino acids of RMF are relatively scattered in the 1–50 region at the N terminus. On the other hand, the sequence and the length of the C-terminal region vary slightly between bacterial species. In order to identify the functional sites in RMF involved in ribosome binding and dimerization, the ability of various RMF mutants to form 100S ribosomes was examined. Table 1 shows the plasmids expressing different RMF mutants. The pRham-02–07 plasmids were designed for the expression of partially deficient RMF lacking 5, 10, and 15 amino acids at the N- or C-terminal region of *E. coli* RMF (Top line in Figure 1 and Supplementary Figure 1). The pRham-08∼20 plasmids are constructed for the expression of RMF substituted conserved amino acids with alanine, in which the first methionine and the twelfth alanine are eliminated. Moreover, the pRham-21–28 plasmids carried RMF constructs in which the four non-conserved amino acids (blue characters in the top line of Figure 1 and Supplementary Figure 1) were deleted or substituted with alanine (Table 1).

The plasmids were transformed into an *E. coli* YB1005 strain lacking the *rmf*, *hpf*, and y*fiA* genes, responsible for 100S ribosome formation (Yoshida and Wada, 2014), and the ability to form the 100S ribosome was examined by the SDGC method.

### Conditions for 100S Ribosome Formation

**FIGURE 2:**
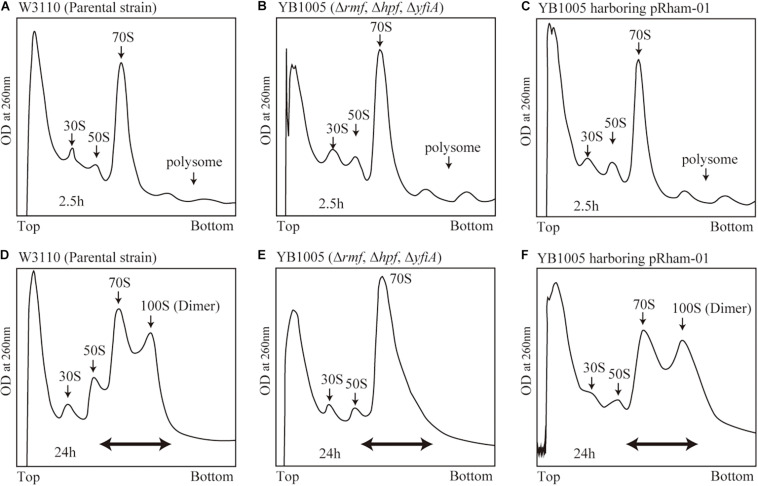
Formation of 100S ribosome after the expression of intact *rmf* and *hpf* genes from the pRham plasmid. **(A–C)** Profiles of ribosomes extracted from the cells during the exponential phase (harvested 2.5 h after inoculation) after sucrose density gradient centrifugation. **(D–F)** Ribosome profiles during the stationary phase (at 24 h). **(A,D)** Represent the W3110 strain (wild-type); **(B,E)** Represent the YB1005 strain (obtained from W3110 by the deletion of *rmf*, *hpf*, and *yfiA* genes); **(C,F)** Represent the YB1005 strain harboring the pRham-01 plasmid (carrying intact *rmf* and *hpf* genes, as shown in Table 1). The double-headed arrows in **(D–F)** indicate the fractionated regions that were analyzed by western blot (see Figure 5).

Obtaining the formation of 100S ribosome by means of plasmid-encoded RMF is a difficult task. RMF that is expressed in the exponential growth phase is rapidly and efficiently degraded (Aiso et al., 2005). Furthermore, during the exponential phase, dimerization of bacterial ribosomes is less likely to occur than during the stationary phase, even in the presence of RMF (Yoshida et al., 2009). Therefore, to efficiently promote dimerization, RMF must be expressed in the stationary phase. However, forcing protein expression in the stationary phase inhibits the formation of 100S ribosomes. Therefore, it is assumed that the timing and strength of RMF expression must be precisely controlled in order to obtain efficient formation of 100S ribosomes. In fact, when exogenous expression of plasmid-encoded RMF was induced by isopropyl β-D-1-thiogalactopyranoside (IPTG) in *E. coli* cells, truncated RMF peptides were observed, and 100S ribosome formation was inefficient (data not shown). To solve this issue, a pRham plasmid for rhamnose-inducible expression was utilized. Since the cAMP receptor protein (CRP), involved in glucose starvation, has been identified as one of the transcription factors regulating *rmf* expression (Shimada et al., 2013), we reasoned that the pRham plasmid could allow for bacterial response to glucose starvation. First, conditions for efficient ribosomal dimerization were identified by changing the treatment timing and rhamnose concentration. As a result, when cells harboring the pRham plasmid were cultivated in a medium containing 0.8% rhamnose for 24 h and harvested, reproducible 100S ribosome formation was obtained to the same extent as that of the wild-type strain (Supplementary Figure 2 and Figures 2D,F). Figure 2A shows a ribosome profile obtained by the SDGC method in W3110 cells during the exponential phase (2.5 h after inoculation). The peaks of the 30S subunit, 50S subunit, 70S ribosome, and polysome were observed. On the other hand, the 100S ribosome was observed instead of the polysome during the stationary phase (24 h after inoculation), as shown in Figure 2D. The 100S ribosome could not be formed in cells with deleted *rmf* and *hpf* genes (YB1005 strain), as shown in Figures 2B,E. When *rmf* and *hpf* gene deficiency was compensated with the pRham plasmid, 100S ribosomes were efficiently formed during the stationary phase (Figure 2F) but not the exponential phase (Figure 2C). Therefore, subsequent experiments were performed under the above-described conditions, i.e., cell cultivation in EP medium containing 0.8% rhamnose, and were harvested 24 h after inoculation.

### Formation of the 100S Ribosome by Mutant RMF

**FIGURE 3:**
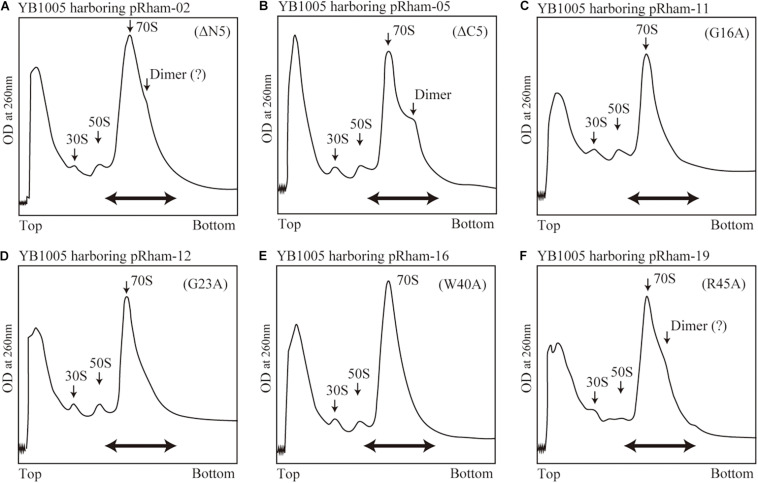
Impact of *rmf* mutations on the formation of 100S ribosomes. The mutant strains shown in Table 1 were cultivated in EP-medium containing 0.8% rhamnose and harvested 24 h after inoculation. The ribosome profiles were analyzed by SDGC method. **(A,B)** Represent the strains expressing RMF truncated by 5 amino acids at N- and C-terminus, respectively; **(C–F)** Represent strains expressing RMF in which the conserved amino acids, glycine (G), cysteine (C), and tryptophan (W), respectively, were substituted with alanine (A) (see Figure 1 and Supplementary Figure 1). The double-headed arrows indicate the fractionated regions that were analyzed by western blot (see Figure 5).

The formation of 100S ribosome (ribosomal dimer) driven by exogenously expressed mutant RMF and intact HPF was analyzed by the SDGC method in *E. coli* cells (YB1005 strain) deleted for *rmf*, *hpf*, and *yfiA* genes (Figure 3 and Supplementary Figure 3). As shown in Figure 2F, 100S ribosomes were efficiently formed upon expression of intact RMF (see also Supplementary Figures 3A1–A3). However, the elimination of five amino acids from the N- or C-terminus of RMF drastically reduced the 100S ribosome formation (Figures 3A,B and Supplementary Figures 3B1–B6). Figures 3C–F show the SDGC patterns obtained when mutant RMF, in which the conserved amino acids had been substituted with alanine, was expressed. Surprisingly, the substitution of one conserved amino acid was sufficient to drastically reduce the 100S ribosome formation (Figures 3C–F and Supplementary Figures 3C1–C13).

**FIGURE 4:**
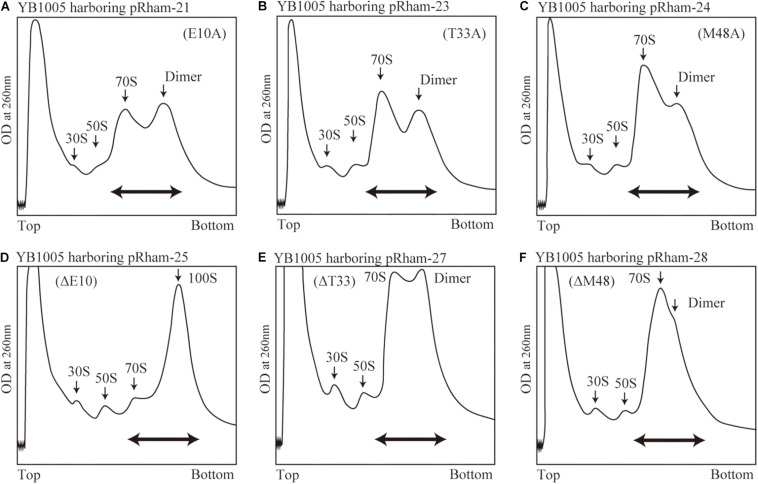
Formation of 100S ribosomes after expression of RMF with mutations involving incompletely conserved amino acids. The experimental procedures are the same as those of Figure 3. **(A–C)** Represent strains expressing RMF in which incompletely conserved amino acids were changed to alanine. **(D–F)** Represent strains expressing RMF deleted for incompletely conserved amino acids. The double-headed arrows in **(A–C)** indicate the fractionated regions that were analyzed by western blot (see Figure 5).

Are all the amino acids in the small protein RMF of *E. coli* essential? To answer this question, mutations affecting incompletely conserved RMF amino acids were inserted (E10, I21, T33, and M48; see Figure 1 and Supplementary Figure 1), and the 100S ribosome formation was examined. Figures 4A,F show the SDGC patterns obtained after substitution and deletion of incompletely conserved amino acids. The substitution of incompletely conserved amino acids does not significantly affect the 100S ribosome formation (Figures 4A–C and Supplementary Figures 3D1–D4), whereas the deletion of E10 (ΔE10) or T33 resulted in enhanced 100S ribosome formation (compare Figures 4D,E and Supplementary Figures 3D5, D7 with Figure 2F). From the above findings, we concluded that the completely conserved RMF amino acids examined in this study were essential for 100S ribosome formation.

### Binding of Mutant RMF and Intact HPF to Ribosomes

**FIGURE 5:**
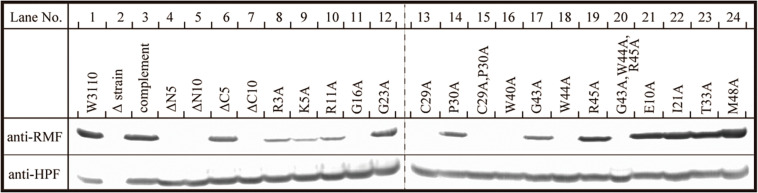
Binding of mutant RMF and intact HPF to ribosomes, analyzed by western blotting. RMF and HPF in ribosomal fractions (see arrows in Figures 2–4) were detected by using the appropriate rabbit antisera. These experiments were repeated as shown in Supplementary Figures 3, 4, and the effects of RMF mutations on dimer formation and ribosome binding are evaluated from these data as shown in Table 2.

The inability of mutant RMF to drive ribosomal dimerization could be either due to the lack of RMF binding to the ribosome or due to its failure to induce the necessary ribosomal conformational changes. To clarify this issue, the binding of mutant RMF to the ribosome was examined by western blotting (Figure 5 and Supplementary Figure 4). Intact RMF and HPF expressed by the pRham-01 plasmid (see Table 1) bound to the ribosome as efficiently as the wild-type strain (lanes 1 and 3 in Figure 5). RMF that is bound to the ribosome is stable, while the unbound RMF is degraded (Wada, 1998). To confirm the mutant RMFs expressed by the plasmids, the detections of RMFs were attempted by western blotting before the strong degradation by use of the harvested cells an hour after induction (Supplementary Figure 5). In Supplementary Figure 5, the bands of some mutant RMFs are not dense, probably because they undergo degradation, but the expressions of all mutant RMF are confirmed. The expressions of HPF shown in Figure 5 support the data that mutant RMFs are expressed, because the expressions of *rmf* and *hpf* genes are carried on the same plasmid. RMF mutations did not affect the binding of intact HPF to the ribosome (lanes 4–24 of “anti-HPF” in Figure 5). As shown in Supplementary Figure 5, the mutant RMFs lacking the 5 and 10 N-terminal amino acids are unstable, which indicates that they are degraded. On the one hand, the mutant RMF lacking the C-terminal 5 amino acids is also unstable, but it, in the ribosomal fraction, is stable in contrast to those lacking the N-terminal amino acids (compare with lanes 4–6 in Supplementary Figure 5 and those in Figure 5). These phenomena can be explained by the fact that RMF bound to ribosomes is stable, while the unbound RMF is degraded. On the other hand, the mutant RMF lacking the C-terminal 10 amino acids is hardly degraded as shown in lane 7 in Supplementary Figure 5. However, this mutant RMF is not able to bind to the ribosome as shown in lane 7 in Figure 5. From these results, it is concluded that the lack of N-terminal 5 (and 10) and C-terminal 10 amino acids inhibited the binding to ribosomes.

The substitution of the conserved G16, C29, W40, or W44 residues with alanine suppressed RMF binding to the ribosome (lanes 11, 13, 16, and 18 of “anti-RMF”). These results explain the lack of 100S ribosomes in Figures 3C,E and Supplementary Figures 3C4,C6,C9,C11. In contrast, the substitution of the conserved R3, K5, R11, G23, P30, G43, or R45 residues with alanine did not affect RMF ability to bind to the ribosome (lanes 8, 9, 10, 12, 14, 17, and 19 of “anti-RMF”). Nevertheless, when these mutated RMF were expressed, the formation of 100S ribosome was nearly abolished as shown in Figures 3D,F and Supplementary Figures 3C1–C3,C5,C7,C10,C12. The substitution of the incompletely conserved E10, I21, T33, or M48 residues with alanine does not affect RMF binding to the ribosome (lanes 21–24 of “anti-RMF”) or 100S ribosome formation (Figures 4A–C and Supplementary Figures 3D1–D4). The effects of RMF mutations on dimer formation and ribosome binding are summarized in Table 2, which are evaluated from the repeated experiments of SDGC and western blotting. Notably, the G23A and R45A mutations impaired the ribosomal dimer formation while not affecting RMF binding to the ribosome.

**TABLE 2:** Evaluation of dimer formation and ribosome binding by RMF.

Mutation	ΔN5	ΔN10	ΔC5	ΔC10	R3A	K5A	R11A	G16A	G23A	C29A	P30A	W40A
Dimer formation	×	×	Δ	×	×	×	×	×	×	×	×	×
Binding to ribosome	×	×	○	×	○	○	○	×	⊚	×	○	×

**Mutation**	**G43A**	**W44A**	**R45A**	**E10A**	**I21A**	**T33A**	**M48A**	**ΔE10**	**ΔI21**	**ΔT33**	**ΔM48**	

Dimer formation	×	×	Δ	⊚	Δ	⊚	⊚	⊚	×	⊚	Δ	
Binding to ribosome	○	×	⊚	⊚	⊚	⊚	⊚	⊚	○	⊚	○	

*The assessment was based on the results of sucrose-density gradient centrifugation (Supplementary Figure 3) and western blotting (Figure 5 and Supplementary Figure 4) analyses. ⊚: Strongly detected, ○: Detected, Δ: Faintly detected, ×: Not detected.*

## Discussion

**FIGURE 6:**
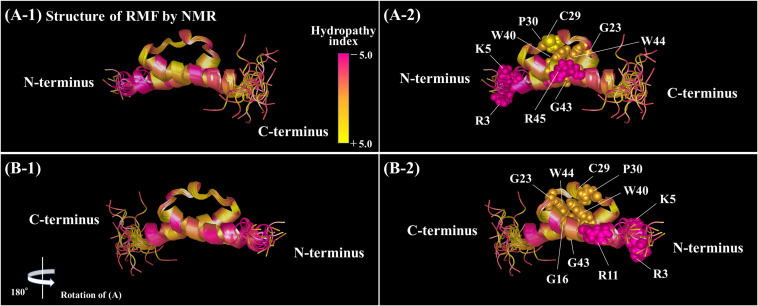
Positions of the conserved amino acids in the structure of RMF from *Vibrio parahaemolyticus* (PDB ID: 2JRM). **(A1,B1)** Are shown in the ribbon model. In **(A2,B2)**, the conserved amino acids are displayed by the ball model. **(B)** is **(A)** rotated by 180° with respect to the vertical axis. The colors represent the hydropathy index. **(A1,B1)** Rotated by 90° with respect to the horizontal axis are shown in Supplementary Figure 6.

The functional sites of RMF were examined using RMF mutants. First, the 100S ribosome formation (dimerization) was assessed by the SDGC method. The substitution of one conserved amino acid with alanine was sufficient to greatly reduce RMF-induced dimerization (Figure 3 and Supplementary Figure 3). In contrast, the replacement of an incompletely conserved amino acid does not affect the 100S ribosome formation (Figures 4A–C). These findings indicated that the conserved RMF amino acids were crucial for 100S ribosome formation. Western blotting analysis revealed that various mutations abolished the ability of RMF to bind to the ribosome (Figure 5 and Supplementary Figure 4). Interestingly, some RMF mutations suppressed ribosomal dimerization without affecting the binding of RMF to the ribosome. Ribosome binding and dimerization abilities of different RMF mutants are summarized in Table 2. The conserved RMF amino acids were mapped on the structure from the NMR (PDB ID: 2JRM) as shown in Figure 6 and Supplementary Figure 6. This structure of *Vibrio parahaemolyticus* RMF is not significantly different from the structure of *E. coli* RMF by the X-ray (PDB ID: 4V8G, Polikanov et al., 2012) or cryo-EM (PDB ID: 6H4N, Beckert et al., 2018; see Supplementary Figure 7). The predicted secondary structure of RMF (Yoshida et al., 2004) was the same as these structures, which consists of two helices and a connecting loop (Figures 6A1,B1). The replacement with alanine of the basic amino acids, R3, K5, or R11, located in the N-terminal domain, resulted in weak binding to the ribosome. However, the removal of the five N-terminal amino acids caused the complete suppression of RMF ability to form 100S ribosomes. Previous cryo-EM structural analysis of *E. coli* 100S ribosomes showed that R3 and K5 interact with helix 28 in 16S rRNA (Beckert et al., 2018). These results suggest that the basic amino acids R3, K5, and R11 in the N-terminal domain may substantially contribute to ribosome binding. Since G16, G23, and G43 are closely located to stabilize the interaction between the two protein helixes, replacing these amino acids might perturb the overall RMF structure (Figures 6A2,B2 and Supplementary Figures 6A2,B2). The mutation of C29 and P30, located in the loop connecting the two helixes, could change the relative position of these structures. Since the larger amino acids, W40 and W44, have parallel indole rings located between the two helices, their substitution with alanine is expected to significantly alter the structure of RMF (Figures 6A2,B2 and Supplementary Figures 6A2,B2). When G23 and R45 were replaced with alanine, ribosome dimerization was strongly impaired despite normal RMF binding to the ribosome (Figures 3, 5 and Table 2), suggesting a role of G23 and R45 in ribosome dimerization. Since the basic residue R45 has been reported to interact with C1536 in the anti-SD (Shine-Dalgarno) sequence of 16S rRNA (Beckert et al., 2018), this interaction may be important for the structural changes required in the 30S subunit for dimerization.

When one of the amino acids that are conserved in all 50 bacterial species examined (“highly conserved”) was replaced with alanine, little or no 100S ribosome formation was observed. In contrast, when the incompletely conserved amino acids were substituted or deleted, RMF binding to ribosomes and formation of 100S ribosomes normally occurred (Figures 4, 5 and Supplementary Figure 4). This highlighted the specificity of the highly conserved amino acids. The deletion of the E10 residue in the N-terminal domain resulted in the formation of an abnormally high number of 100S ribosomes (Figure 4D). The N-terminal basic amino acids, R3, K5, and R11, are presumed to contribute significantly to ribosome binding. Consistently, deletion of the acidic E10 in the N-terminal domain promoted the formation of 100S ribosomes by increasing RMF stability and binding to the ribosomes.

In summary, the conserved amino acids in RMF were found to play a role in the conformational and stability changes required for ribosome binding and dimerization, whereas the incompletely conserved amino acids may regulate the binding activity of RMF to the ribosome. These data provide new insights into the conformational changes faced by bacterial ribosomes and may contribute to the development of antibiotic or bacteriostatic agents.

## Data Availability Statement

The raw data supporting the conclusions of this article will be made available by the authors, without undue reservation.

## Author Contributions

HY and AW conceived the study and designed the experiments. HY performed most of the experiments. MU and CW provided the mutant cells. HN and YM contributed to the construction of plasmids. All authors contributed to the article and approved the submitted version.

## Conflict of Interest

HN was employed by the company HORIBA, Ltd. The remaining authors declare that the research was conducted in the absence of any commercial or financial relationships that could be construed as a potential conflict of interest.

## Supplementary Material

Supplementary Figure 1 Alignment of RMF amino acid sequences between 50 species of gammaproteobacteria. Completely conserved amino acids are represented in red. RMF of the *E. coli* strain used in this work is shown at the top with a yellow background. The representative pathogens, *Yersinia pestis* and *Vibrio cholerae*, are indicated by blue background. The structure of RMF (PDB ID: 2JRM) in Figure 6 and Supplementary Figure 6 is that of *Vibrio parahaemolyticus*, which is shown with a green background. Incompletely conserved amino acids were used as references and are shown in blue characters in the *E. coli* sequence (top line).

Supplementary Figure 2 Optimization of conditions for ribosomal dimerization after the expression of *rmf* and *hpf* genes from plasmids. YB1005 cells harboring the pRham-01 plasmid (see Table 1), cultivated in a medium containing 0.4% **(A–C)** or 0.8% **(D–F)** rhamnose, were harvested after 6 h **(A,D)**, 9 h **(B,E)**, or 24 h **(C,F)** of culture. The ribosome profiles were analyzed by the SDGC method.

Supplementary Figure 3 Ribosome profiles of the strains harboring the plasmids shown in Table 1. **(A1,A2)** Represent parental and mutated strain lacking key factors for 100S ribosome formation, respectively. **(A3)** Represents strain expressing intact *rmf* and *hpf* genes. **(B1–B6)** Represent strains expressing RMF with N- or C-terminal truncations. **(C1–C13)** Represent strains in which the conserved amino acids RMF were replaced by alanine (A). **(D1–D8)** Represent strains expressing RMF mutated for incompletely conserved amino acids. Figures 2–4 refer to these data. The double-headed arrows indicate the fractionated regions (① 70S and ② 100S ribosomal fractions) that were analyzed by western blot (see Supplementary Figure 4).

Supplementary Figure 4 Western blotting analysis of mutant RMF and intact HPF binding to ribosomes. RMF and HPF in the 70S and 100S ribosomal fractions (see double-headed arrows in Supplementary Figure 3) were detected using the appropriate rabbit antisera. Each experiment was conducted multiple times. **(A1–B4)** Represent detection by anti-RMF antibodies. These results were used to determine the efficiency of ribosome binding, as shown in Table 2. **(C1–C4)** Represent detection by anti-HPF antibodies.

Supplementary Figure 5 The mutant RMFs were detected using the appropriate rabbit antisera before the strong degradation by use of the harvested cells an hour after induction. RplB (ribosomal protein L2) was also detected for reference.

Supplementary Figure 6 Position of the conserved amino acids in the structure of RMF from *Vibrio parahaemolyticus* (PDB ID: 2JRM). **(A1,B1)** Are shown in the ribbon model. In **(A2,B2)**, the conserved amino acids are displayed by the ball model. The colors represent the hydropathy index. **(A,B)** Represent the amino acids shown in Figure 6, rotated by 90° with respect to the horizontal axis.

Supplementary Figure 7 Structural superposition of RMF from NMR (PDB ID: 2JRM, Magenta), X-ray (PDB ID: 4V8G, Blue), and Cryo-EM (PDB ID: 6H4N, Green).
