# Supplementary material for: Functional Sites of Ribosome Modulation Factor (RMF) Involved in the Formation of 100S Ribosome
Source: Front Mol Biosci. 2021 May 3;8:661691. doi: 10.3389/fmolb.2021.661691 (PMC8126665; doi:10.3389/fmolb.2021.661691)
Supplement: Supplementary Figure 1 — Alignment of RMF amino acid sequences between 50 species of gammaproteobacteria. Completely conserved amino acids are represented in red. RMF of the E. coli strain used in this work is shown at the top with a yellow background. The representative pathogens, Yersinia pestis and Vibrio cholerae, are indicated by blue background. The structure of RMF (PDB ID: 2JRM) in Figure 6 and Supplementary Figure 6 is that of Vibrio parahaemolyticus, which is shown with a green background. Incompletely conserved amino acids were used as references and are shown in blue characters in the E. coli sequence (top line). [file Data_Sheet_1.PDF]

Figure S1

Bacterial species	Amino acid sequences of RMF
Escherichia coli K-12	MKRQKRDRLERAHQRGYQAGIAGRSKEMCPYQTLNQRSQWLGGWREAMADRVVMA
Shigella sonnei	MKRQKRDRLERAHQRGYQAGIAGRSKEMCPYQTLNQRSQWLGGWREAMADRVVMA
Citrobacter rodentium	MKRQKRDRLERAHQRGYQAGIAGRSKEMCPYQTLNQRSHWLGGWREAMADRVVMA
Enterobacter asburiae	MKRQKRDRLERAHQRGYQAGIAGRSKEMCPYQTINQRSQWLGGWREAIGDRVLIA
Salmonella enterica	MKRQKRDRLERAHQRGYQAGIAGRSKEMCPYQTLNQRSYWLGGWRQAMEDRAVMA
Klebsiella pneumoniae	MKRQKRDRLERAHQRGYQAGIAGRSKEMCPYQSLNQRSWWLGGWREAMEDRVLTA
Enterobacteriaceae bacterium	MKRQKRDRLERAHQRGYQAGIAGKSKEICPYQTLNQRSHWLGGWREAMEDRTVIA
Kosakonia sacchari	MKRQKRDRLERAHQRGYQAGITGRPKEMCPYQTLNQRSEWLGGWREAMEDRAVIA
Pluralibacter gergoviae	MKRQKRDRLERAHQRGYQAGIAGKSKEICPYQTINQRSHWLGGWREAMEDRAVIA
Raoultella ornithinolytica	MKRQKRDRLERAHQRGYQAGITGRSKEMCPYQTLNQRSYWLGGWREAMEDRVQTA
Cronobacter sakazakii	MKRQKRDRLERAHHRGYQAGITGRSKEMCPYQTLNQRSYWLGGWREAMEDRAQIA
Siccibacter turicensis	MKRQKRDRLERAHHRGYQAGITGRSKEMCPYQTLNQRSYWLGGWREAMEDRAQIA
Shimwellia blattae	MKRQKRDRLERAHQRGYQAGITGRSKEMCPYQALTQRSHWLGGWRKAMEDRVAIA
Dickeya dadantii	MKRQKRDRLARAHSRGYQAGIIGRSKELCPYQSIDARSNWLGGWREAMEDRAVMA
Pantoea vagans	MKRQKRDRLERAHSRGYQAGITGRSKEMCPYQLIDAKSHWLGGWRQAMEDRSVAA
Erwinia amylovora	MKRQKRDRLDRAHSRGYHAGITGRSKEMCPYQLLDARSHWLGGWRQAMEDRTVTA
Pectobacterium wasabiae	MKRQKRDRLERAHSRGYQAGIVGRPKEFCPYQSINARSYWLGGWRKAMEDRAVTA
Rahnella aquatilis	MKRQKRDRLERAQSRGYQAGIGGRSKEICPYQSLDARSHWLGGWRQAMEDRAVTA
Providencia stuartii	MKRQKRDRLERALSRGYQAGIAGRSKELCPYQSVDARSHWLGGWRKAMEDRAILSA
Serratia marcescens	MKRQKRDRLERAHSRGYQAGILGRSREHCPYQSSVDARSQWLGGWREAMEDRAVTA
Candidatus Sodalis pierantonius	MKRQKRDRLERAHSRGYQAGLSGRPRELCPYQSLDARSHWLGGWRKAMEDRSVI
Yersinia pestis	MKRQKRDRLERALSRGYQAGISGRSREICPYQSLDARSHWLGGWRQAMEDRAVTA
Edwardsiella piscicida	MKRQKRDRLERAQARGYQAGIAGRSKELCPYHSLDARSYWLGGWRQAMEDRSLVAVA
Paraglaciecola psychrophila	MKRQKRDKLSRAHSKGYQAGITGRSKEHCPFQSTNVRSEWLGGWREAIGDRSIGLASR
Alteromonas macleodii	MKRQKRDKLTRAHSKGYQAGISGRSKENCPFQQNDARSQWLGGWREAVEDRHLGLSVK
Pseudoalteromonas haloplanktis	MKRQKRDRLERAHSQGFKAGLAGRSKEHCPYQQVDPKSEWLGGWREAIDNRNIYKT
Photorhabdus luminescens	MKRQKRDRLARALSRGYHAGILGRPREHCPYYSLDARSHWLGGWRQAMEDRAMMA
Shewanella amazonensis	MKRQKRDRLDRAFAKGFQAGVGGRSKEMCPYSNLDSRSQWLGGWREGVDGRITGLFTK
Colwellia psychrerythraea	MKRQKRDRQSRAHTRGYQAGISGRSKEHCPYQIDAIKSQWLGGWREAIEDKQQGLFK
Tolumonas auensis	MKRQKRDRLERARTKGYQAGLAGRSKELCPYQCIDARGYWLGGWRDAVDERSQGYMVN
Xenorhabdus bovienii	MKRQKRDRLARALSRGYQAGIHGRPRENCPYHSLDARSHWLGGWRQAMEDRA
Aeromonas salmonicida	MKRQKRDRLERARARGYQAGVVGKQKEACPYQCLDARGHWLGGWRDAMEGRGSGLFMK
Moritella viscosa	MKRQKRDRLERAYSKGYQAGAAGRSNEVCPFQSADSRGQWLGGWREAQEIRSTGLFMK
Proteus mirabilis	MKRQKRDRLARALSKGYQAGMQGRSKEQCPYFAIDARSHWLGGWRQAMEDRPGLAK
Aliivibrio salmonicida	MKRQKRDRLERAQSQGYKAGINGRSSENCPYQTMDARSCWLGGWRDARDDKQSGLFK
Idiomarina loihiensis	MKRQKRDRFERAHTQGFKAGLHGRSKDNCPYQTQDDFRSHWLGGWRDAMEARNTGLFR
Vibrio parahaemolyticus	MKRQKRDRLERAQSQGYKAGLNGRSQEACPYQQVDARSYWLGGWRDARDEKQSGLYK
Vibrio cholerae	MKRQKRDRLERAQSQGYKAGLNGRSHDECPYQQTEVRSYWLGGWRDARNDKLSGLCK
Ferrimonas balearica	MKRQKRDKMDRAFSRGFQAGLTGRSKENCPYHAPDAKMHWLGGWREAIDGRETGLFTK
Kangiella koreensis	MKRQKRDKLQRAHTKGFNAALQGQSKENCPFEELNAREEWLGGWREGREAFTTNGMKAYI
Alcanivorax pacificus	MKRQKRDRTNRAYSRGYHAGLHGKSQDLCPFDALMSRSSWINGWREGRTDHINGYVGVAS
Cellvibrio japonicus	MKRQKRDQTERAFVKGYQAGIEGRSKSLCPHESGLARQQWLNGWRESRMDQWDGYSRLAQ
Halomonas campaniensis	MKRQKRDRFQRAYVHGYKAGITGRSRDDCPSQDVNLREYWMSGWREGRGDQWDGMTGVSGIHKNPMVMT
Chromohalobacter salexigens	MKRQKRDRFQRAYVHGYKAGMTGRSRDDCPSQDINLREYWMSGWREGRGDQWAGMTGISGIHKNPMVLS
Hahella chejuensis	MRRQKRDMFERAYLKGYRAGVSGRSKDLCPAANPQMRQEWINGWRDGRADHWDGYTGVSGIHRSPGIAS
Gynuella sunshinyii	MRRQKRDRMERAFQKGYMAGVEGKSKDSCPKVNPTEHQEWLNGWREGRVDNWDGLTGVRGIHRIAEITMR
Saccharophagus degradans	MKRQKRDHTSRAFTRGYQAGVEGRSRSLCPHSTGEIKQSWLTGWREGREDHWNGFNTLAQVQRISNIS
Pseudomonas putida	MRRLKRDPLERAYSRGYQYGVTGKSRELCPFNLPSVRQAWINGWREGRGDNWDGMTGTAGIHRLNENHAVG
Teredinibacter turnerae	MKRQKKNSSQRAFSRGYQAGVTGRSRSLCPHVTGDTRHDWLTGWREGREDHWNGFNTLAQAQKIQTFF
Azotobacter chroococcum	MRRLKRDPLERAFLRGYQNGIHGKSRELCPFTHPSTRQAWINGWREGRGDNWDGLTGTAGIHRLNQLRAVS
Marinobacter hydrocarbonoclasticus	MKRQKRDMYARAFKRGYLAGVSGKSKDSCPLEQPEVRQEWLNGWREGRTDNWEGMTGVSGIHKLANVTAT
