# Supplementary figures and images for: Functional Sites of Ribosome Modulation Factor (RMF) Involved in the Formation of 100S Ribosome
Source: Front Mol Biosci. 2021 May 3;8:661691. doi: 10.3389/fmolb.2021.661691 (PMC8126665; doi:10.3389/fmolb.2021.661691)

Supplementary Figure 2

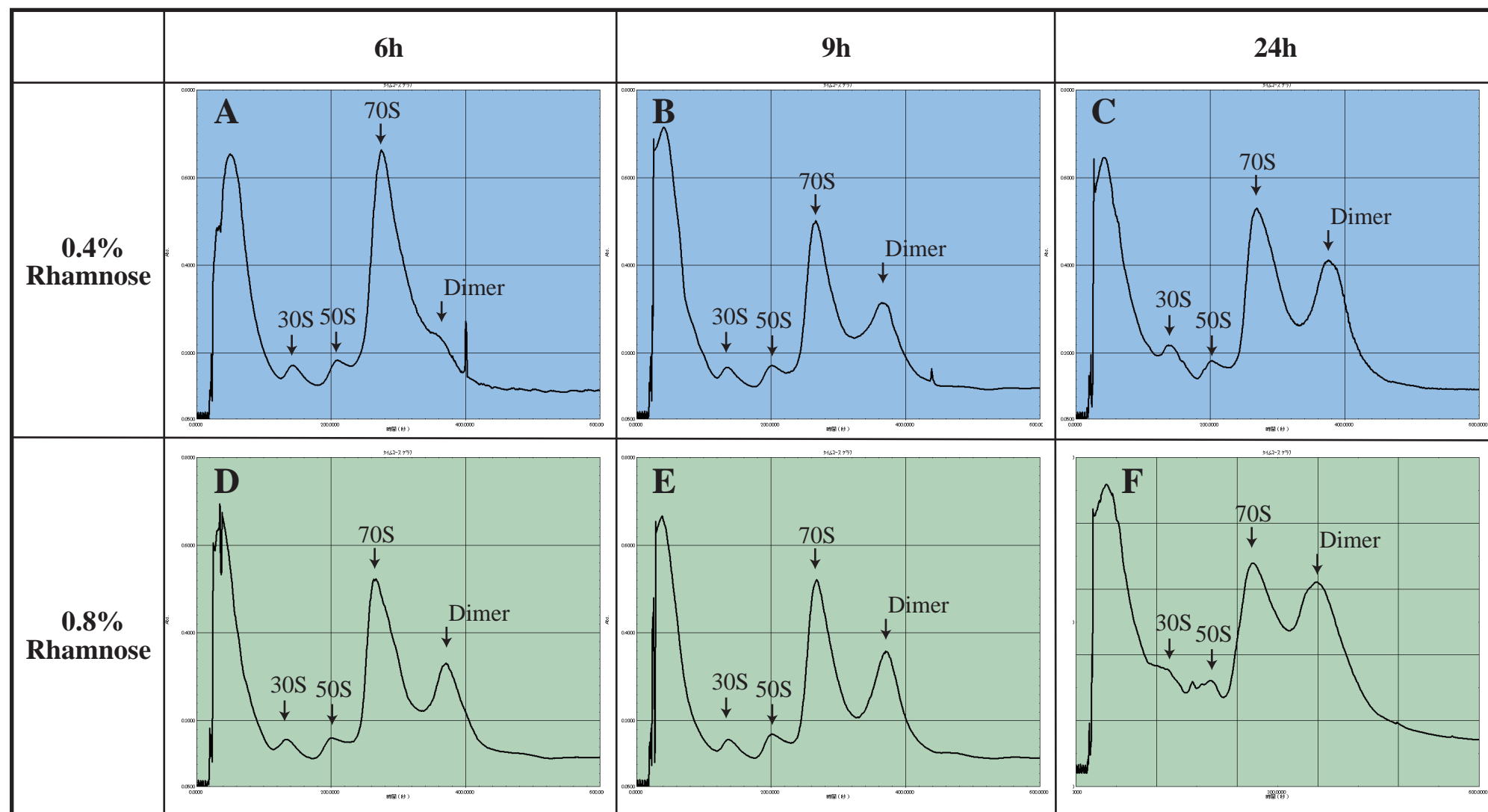

Supplement: Supplementary Figure 2 — Optimization of conditions for ribosomal dimerization after the expression of rmf and hpf genes from plasmids. YB1005 cells harboring the pRham-01 plasmid (see Table 1), cultivated in a medium containing 0.4% (A–C) or 0.8% (D–F) rhamnose, were harvested after 6 h (A,D), 9 h (B,E), or 24 h (C,F) of culture. The ribosome profiles were analyzed by the SDGC method. [file Data_Sheet_2.PDF]

Supplementary Figure 3

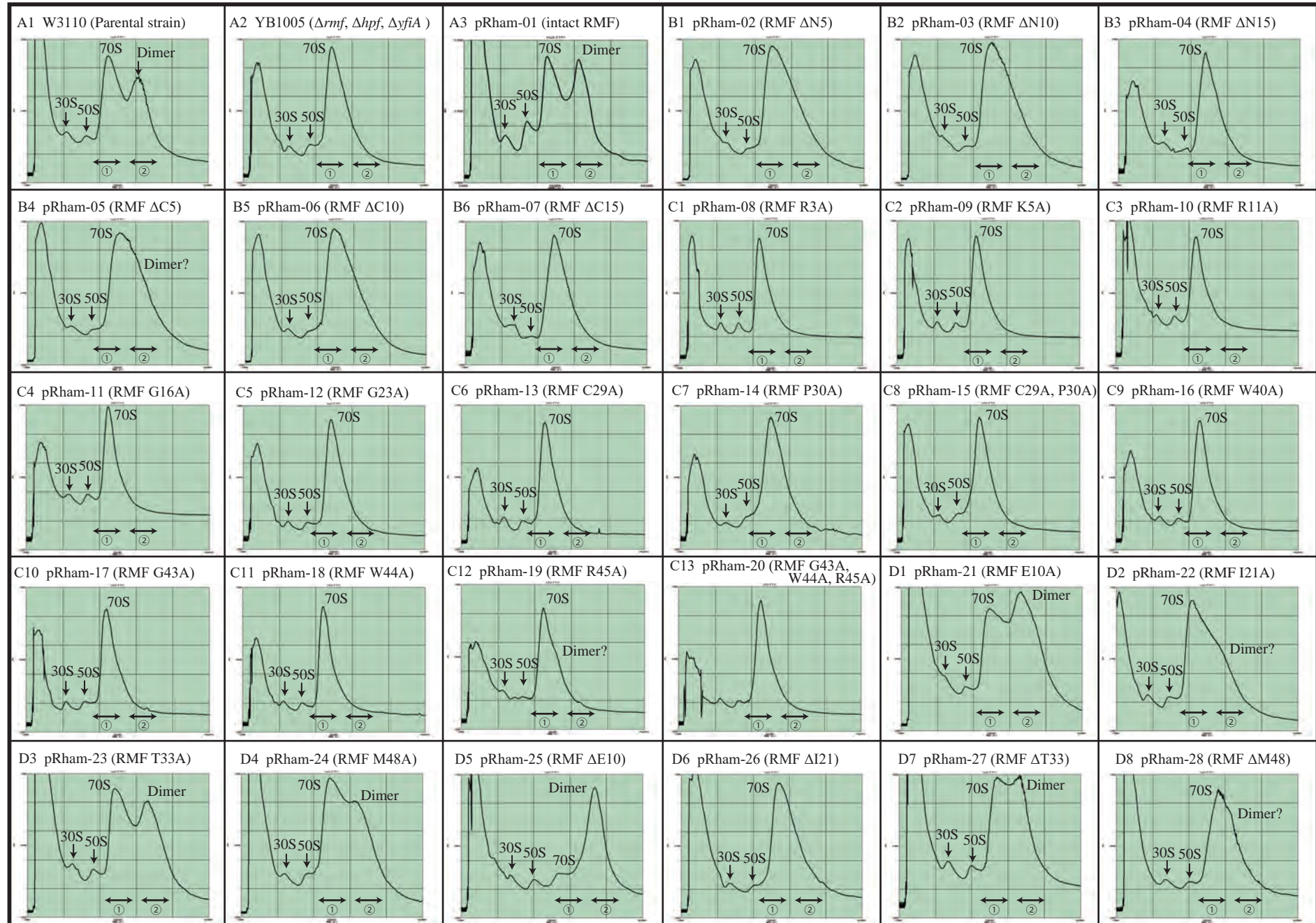

Supplement: Supplementary Figure 3 — Ribosome profiles of the strains harboring the plasmids shown in Table 1. (A1,A2) Represent parental and mutated strain lacking key factors for 100S ribosome formation, respectively. (A3) Represents strain expressing intact rmf and hpf genes. (B1–B6) Represent strains expressing RMF with N- or C-terminal truncations. (C1–C13) Represent strains in which the conserved amino acids RMF were replaced by alanine (A). (D1–D8) Represent strains expressing RMF mutated for incompletely conserved amino acids. Figures 2–4 refer to these data. The double-headed arrows indicate the fractionated regions (① 70S and ② 100S ribosomal fractions) that were analyzed by western blot (see Supplementary Figure 4). [file Data_Sheet_3.PDF]

Supplementary Figure 4

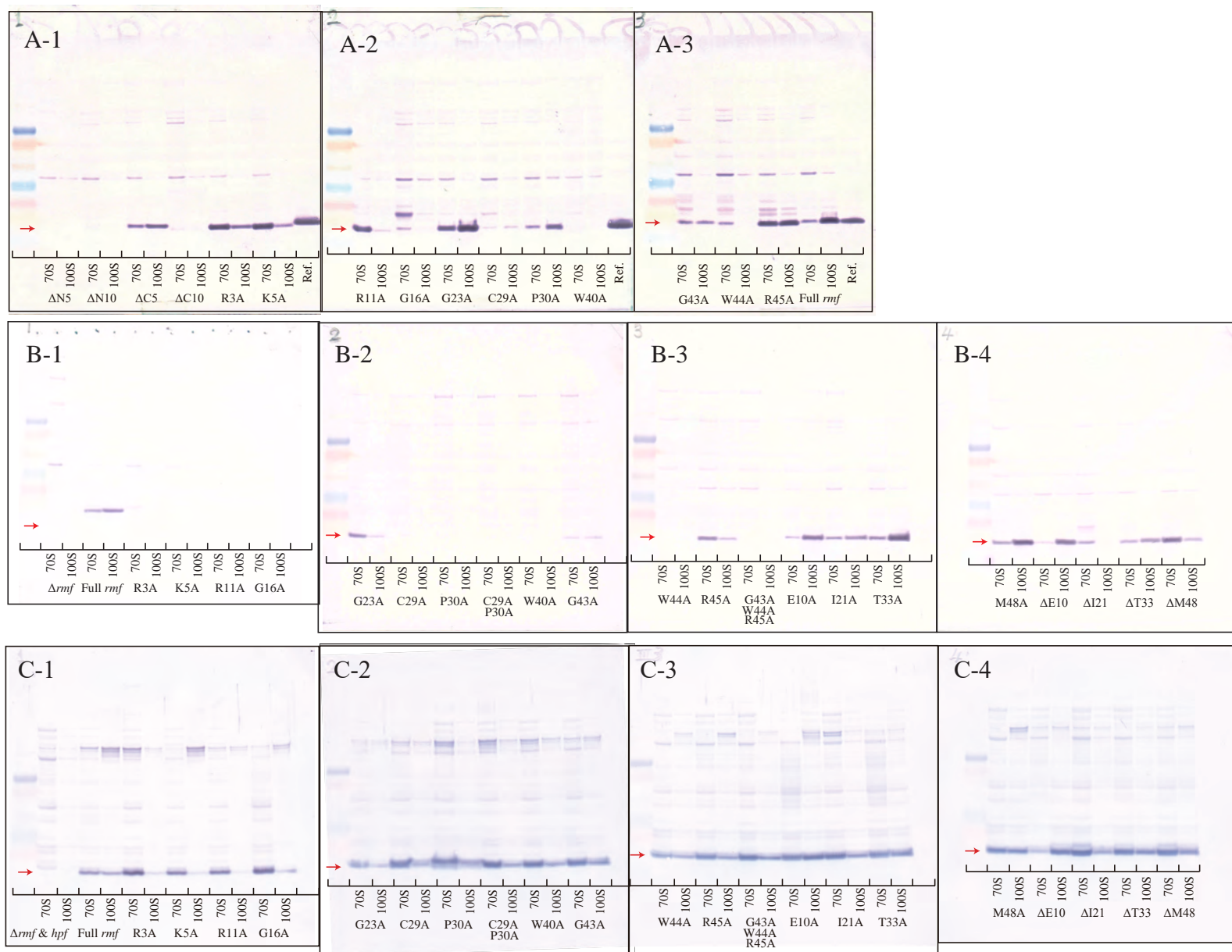

Supplement: Supplementary Figure 4 — Western blotting analysis of mutant RMF and intact HPF binding to ribosomes. RMF and HPF in the 70S and 100S ribosomal fractions (see double-headed arrows in Supplementary Figure 3) were detected using the appropriate rabbit antisera. Each experiment was conducted multiple times. (A1–B4) Represent detection by anti-RMF antibodies. These results were used to determine the efficiency of ribosome binding, as shown in Table 2. (C1–C4) Represent detection by anti-HPF antibodies. [file Data_Sheet_4.PDF]

Supplementary Figure 5

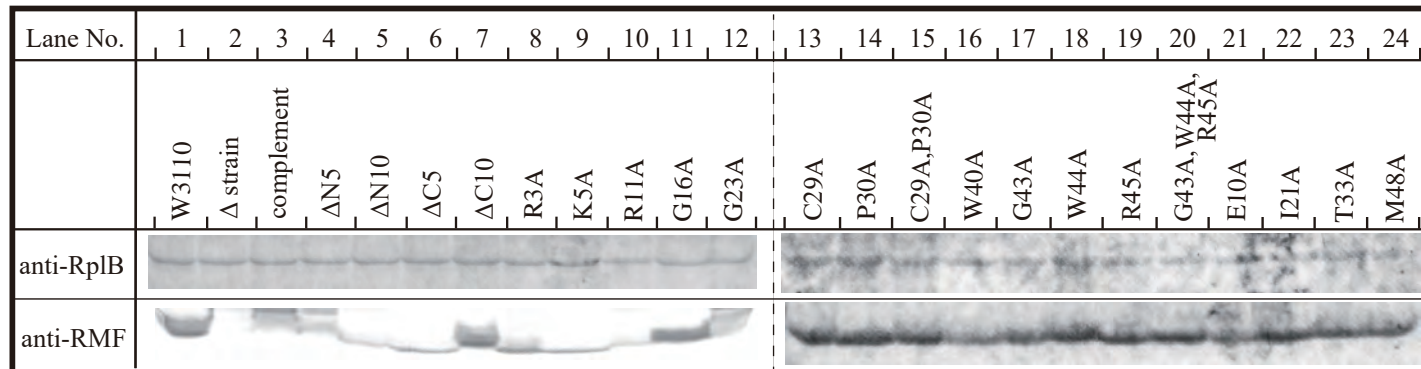

Supplement: Supplementary Figure 5 — The mutant RMFs were detected using the appropriate rabbit antisera before the strong degradation by use of the harvested cells an hour after induction. RplB (ribosomal protein L2) was also detected for reference. [file Data_Sheet_5.PDF]

Supplementary Figure 6

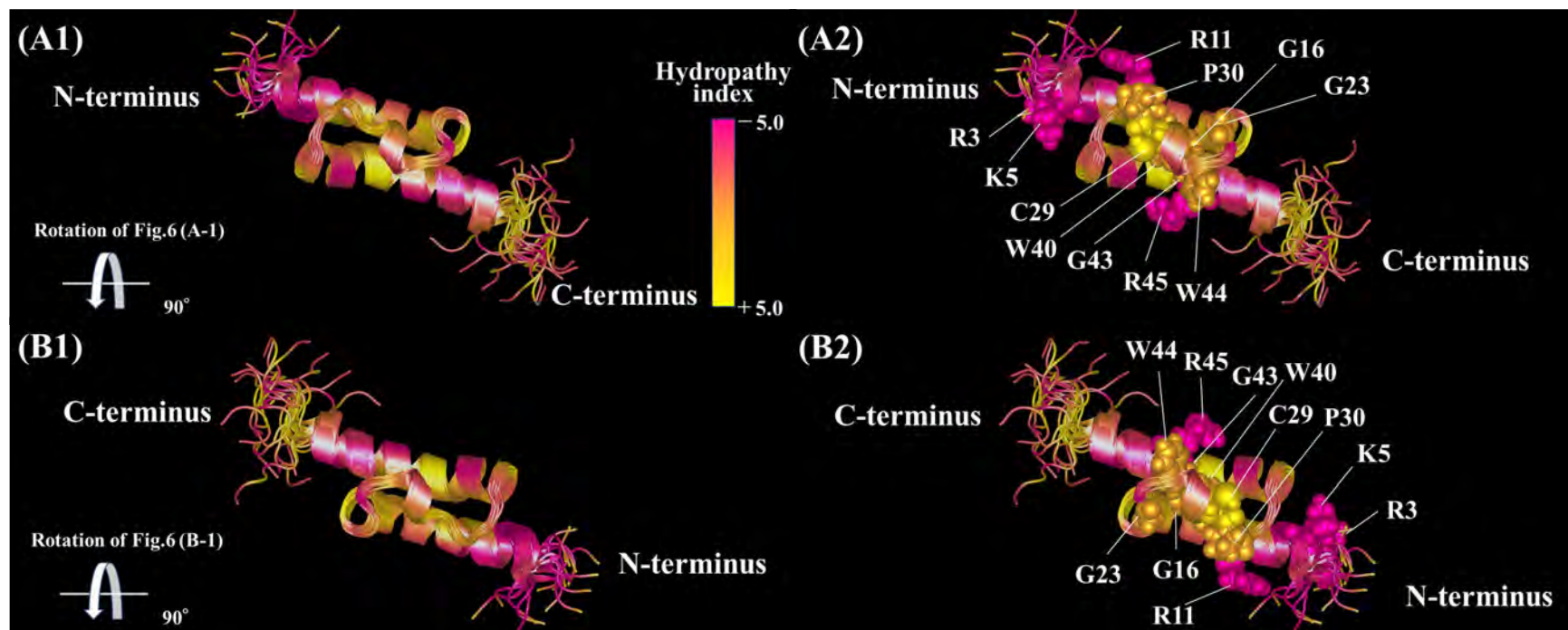

Supplement: Supplementary Figure 6 — Position of the conserved amino acids in the structure of RMF from Vibrio parahaemolyticus (PDB ID: 2JRM). (A1,B1) Are shown in the ribbon model. In (A2,B2), the conserved amino acids are displayed by the ball model. The colors represent the hydropathy index. (A,B) Represent the amino acids shown in Figure 6, rotated by 90° with respect to the horizontal axis. [file Data_Sheet_6.PDF]

## Supplementary Figure 7

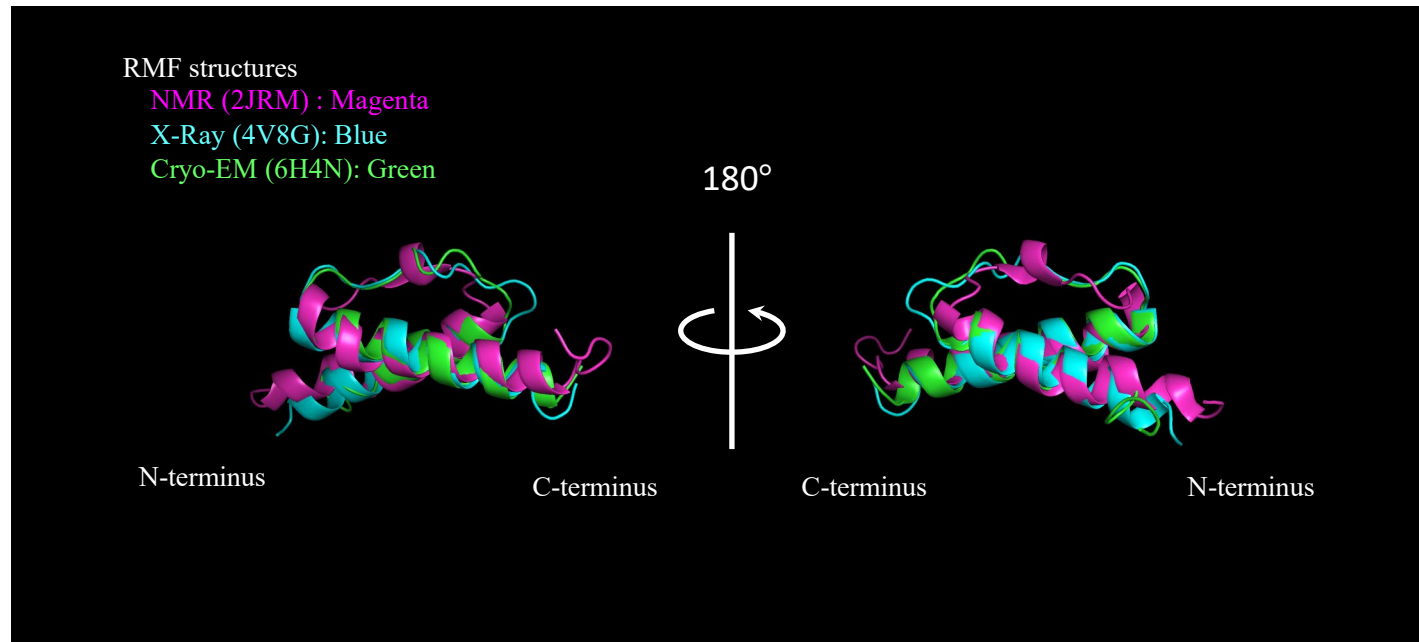

Supplement: Supplementary Figure 7 — Structural superposition of RMF from NMR (PDB ID: 2JRM, Magenta), X-ray (PDB ID: 4V8G, Blue), and Cryo-EM (PDB ID: 6H4N, Green). [file Data_Sheet_7.pdf]
